# Reliability and validity of “My Jump 2” application for countermovement jump free arm and interlimb jump symmetry in different sports of professional athletes

**DOI:** 10.7717/peerj.17658

**Published:** 2024-07-09

**Authors:** Yong Peng, Shaotong Sun, Yudi Wang, Ya xuan Qin, Di Qin

**Affiliations:** 1 Jiangsu Collaborative Innovation Center for Sports and Health Project, Nanjing Sport Institute, Nanjing, China; 2 Key Laboratory of Human Sports Science for Jiangsu Province, Nanjing Sport Institute, Nanjing, China; 3 School of Sport Health, Nanjing Sport Institute, Nanjing, China; 4 School of Physical Education and Nursing, Chengdu College of Arts and Sciences, Chengdu, China

**Keywords:** My jump, Vertical jump, Athletes, Reliability, Validity

## Abstract

**Background:**

Vertical jumping is an important evaluation tool to measure muscle strength and power as well as lower limb symmetry. It is of practical importance and value to develop and utilize a portable and low-cost mobile application (APP) to evaluate jumping. The “My Jump 2” app is an iPhone camera-based application for measuring jumping movements, which is applied to the countermovement jump (CMJ) vertical jumps of the lower limbs of athletes in different sports. The validity of this application and previous versions applied to different forms of vertical jump tests has been preliminarily demonstrated in different population, which has an obvious progress in research. Therefore, the reliability and validity of the jump height, time of flight parameters and symmetry of the CMJ vertical jump of athletes in different sports are needed to be verified by more experiments.

**Purpose:**

The purpose of this study is to verify whether “My Jump 2” can effectively and reliably assess jump height, flight practice and lower limb symmetry in CMJAM (countermovement jump free arm) tests in fencing, swimming and diving athletes.

**Methods:**

Seventy-nine fencers, swimmers and divers with training experience participated in this study. They completed a total of three CMJAM vertical jump and lower limb symmetry tests in 1 day, while being assessed by using the “My Jump 2” application and a force platform. The intra-group correlation coefficient (ICC) was used to verify reliability, while the Cronbach’s alpha and coefficient of variation (CV%) was used to analyze the stability of the CMJAM vertical jump test over three jumps. The Pearson correlation coefficient was used to verify the strength of the relationship between methods (*i.e*., concurrent validity), and the Bland-Altman plot was used to represent consistency, meanwhile, the t-test was used to determine the systematic bias between methods.

**Results:**

Compared with the force platform, the cumulative height values of the total number of jumps (r = 0.999; *p* = 0.000), the cumulative time to vacate (r = 0.999; *p* = 0.000) for the CMJAM test obtained by the “My Jump 2” application, the height (ICC = 0.999–1, *p* = 0.000), the time to vacate flight (ICC = 0.999–1, *p* = 0.000), contact time symmetry (ICC = 0.976–0.994, *p* = 0.000), and flight time symmetry (ICC = 0.921–0.982, *p* = 0.000), respectively. Showed high correlation between the results of “my jump 2” app and the force platform.

**Conclusion:**

The “My Jump 2” application is a valid tool to assess CMJAM vertical jump and lower limb symmetry in fencing, swimming and diving athletes with training experience.

## Introduction

Jumping ability (explosive power) is a crucial sports ability for successful completion of multi-sport events, which has been proved to correlate with athletes’ performance in competition (Prieske et al., 2019; Rodríguez-Rosell et al., 2017). The incorrect jumping posture may cause orthopedic diseases such as muscle strain, joint dislocation and even fracture of the lower limbs. Therefore, assessing jumping ability can not only predict the risk of lower limb injury, but also serve as a reference for athlete selection (Arede et al., 2019; Lehance et al., 2009). Vertical jump performance is related to muscle strength, neuromuscular fatigue and motor performance of lower limb in physical conditioning training. It’s the most common method to measure lower explosive motor performance, which includes different vertical jumps such as static squat jumps, reverse motion jumps and deep jump exercises. Jumping performance has been widely used to assess muscle strength, power and limbs asymmetry. Asymmetry is an indicator of muscle and joint imbalance, which is considered an important indicator of injury prevention. The studies showed that the injury rate of the non-dominant side could reach 2.3 times more likely in the presence of lower limb asymmetry (Attwood et al., 2019). The injury rate of the non-dominant side can multiply when the muscle strength asymmetry on both sides of the lower limb reaches 10% or more (Orchard et al., 1997). The imbalance of lower limb strength will make the non-dominant limb become weak part in the movement and increase the risk of injury. Lower limb asymmetry directly affects the athlete’ s strength, explosive power, multidirectional speed, balance ability and so on (Bishop et al., 2021; Kotsifaki et al., 2023). Insufficient strength and large asymmetrical differences in the muscles of the lower limb will have a negative impact on the performance ability and special performance improvement in athletes, which may lead to sports injury. Therefore, jumping is an important tool for assessing muscle strength and power as well as interlimb symmetry.

Force platform have been considered the gold standard measurement tool for assessing vertical jump height for a long time. In addition, the infrared platform, camera, accelerometer and other tools are also verified as effective tools to measure vertical jump performance (Stevenson et al., 2015). However, the above measurements are usually laboratory tests, expensive, limited equipment, large volume, poor portability, and a complex testing process, which also needs a perform specific computer software. Based on the above problems, further search for simpler, more accurate and reliable assessment methods is urgently needed.

Recently, an application called “My Jump 2” claims to calculate the height of a jump by recording high-speed video directly with the iPhone’s 240 Hz camera. The application is inexpensive, practical and can be used in different areas of sports. This technology is not only used to monitor the physical fitness level among the general population, but also to evaluate the physical conditioning of professional athlete. The validity of this application and previous versions applied to different forms of vertical jump tests has been preliminarily demonstrated in groups of soccer players (Barbalho et al., 2021), professional sprint distance runners and throwers (Gallardo-Fuentes et al., 2016), youth basketball players (Bishop et al., 2022), soccer players with cerebral palsy (Coswig et al., 2019), elementary school students (Bogataj et al., 2020), sports science students (Haynes et al., 2019), and older adults (Cruvinel-Cabral et al., 2018). The current study included a relatively small sample of athletes with training experience. However, the application of “My Jump 2” has not been verified for fencing, swimming, diving and other athletes with training experience, especially the lack of vertical jump action mode in swimming, fencing and other special sports. Considering that the reliability of the vertical jump explosive strength test has different gender, training experience and different specific training populations, and the reliability and effectiveness of My jump 2 have not been validated in athletes of Chinese athletes. So as to provide a richer basis for the application of this application to test jump explosive power in a wide group of athletes, it is necessary to study and validate the validity and reliability of this application in athletes of Chinese descent and athletes of more sports except track and field, soccer and basketball.

Therefore, we assume that the “My Jump 2” application is a valid and reliable tool that can be used to evaluate vertical jumps for multiple groups of athletes tested in fencing, swimming, diving, *etc*. This study intends to verify the validity of the “My Jump 2” application in assessing vertical jumping ability in different professional athletes by comparing it with the consistency of the countermovement Jump free arm (CMJAM) test and the asymmetry test of the lower limbs in a group of fencing, swimming and diving athletes with long training experience.

## Methods

### Experimental protocols

After becoming familiar with the basic test process and learning the test movements, athletes performed CMJAM and lower limb symmetry test three times on the same day with best efforts. The “My Jump 2” application and the Kistler 3D force table were used to measure the jump height and flight time of the CMJAM test, and to measure the contact time, flight time, flight time symmetry and contact time symmetry of the lower limb symmetry test. The above parameters obtained from both tests were used for simultaneous validity analysis.

### Subjects

The recruitment period of this experiment started at June 20th and ended at August 31st in 2022. A total of 29 athletes with training experience are included in this experiment, 12 of whom are from the Jiangsu fencing team (male *n* = 6, female *n* = 6), 10 of whom are from the swimming team (male *n* = 8, female *n* = 2), 7 of whom are from the diving team (male *n* = 2, female *n* = 5). The specific basic information of all subjects can be found in Table 1. Before the test, the subjects were informed of the specific procedure of the experiment and the risks and benefits associated with participating in the experiment. The informed consent documents were signed and obtained the ethical approval (RT-2022-05) of the ethics committee of Nanjing Sports Institute before the test.

**Table 1:** Basic information of subjects.

Basic data	Fencing (Male, *n* = 6)	Fencing (Female, *n* = 6)	Swimming/diving (Male, *n* = 10)	Swimming/diving (Female, *n* = 7)
Age (Year)	20.50 ± 1.76 (18 to 23)	21.00 ± 3.03 (18 to 27)	13.7 ± 1.64 (13 to 18)	12.86 ± 1.46 (11 to 16)
Height (cm)	181.83 ± 3.43	169.00 ± 5.93	171.50 ± 15.84	149.43 ± 13.16
Weight (kg)	70.88 ± 7.77	60.50 ± 8.59	55.74 ± 14.05	40.33 ± 12.08
Years of training (year)	9.67 ± 1.75	9.50 ± 4.09	6.8 ± 0.63	7.43 ± 1.72

Exclusion criteria: 1. Medical history of lower extremity joint injuries or low back injuries and other potential medical problems in the three months before participating in the trial; 2. Medical or orthopedic problems that can affect their participation in the study; 3. Any lower extremity reconstructive surgery or unresolved neuromuscular-skeletal disease within the past 2 years; 4. Taking or having taken steroids, growth hormones, or any type of related motor skill enhancing drugs (except natural supplements such as vitamins and minerals); 5. With less than two years of competitive competition experience or less than 10 h of training per week.

### Experimental equipment

The iPhone application “My Jump 2” is an application for calculating the height of jumps, originally developed with Xcode software (5.0.5 for Mac OSX 10.9.2; iPhone Inc., Cupertino, CA, USA). The application is installed on the iPhone 13ProMax equipped with a 240 Hz high-speed camera with 720 p recording quality. Designed for analyzing vertical jumps, the application allows the user to select the time (in ms) between two frames. Subsequently, the formula H = t^2^ * 1.22625 is used to calculate the height of the jump.

The flight time and altitude of CMJAM and drop jump (DJ) are measured by a 3D force measuring table (9260AA; Kistler, Winterthur, Switzerland), and the motion analysis and data processing are proceeded by Kistler’s MARS software, while the jump motion images are recorded on iPhone 13 ProMax for editing by “My Jump 2” app software.

### Procedures

All experiments were completed in the Sports biomechanics Laboratory of Nanjing Sports Institute. The experimenters uploaded the individual information of all the subjects at the application “My Jump 2” to ensure all the data can be corresponded to the ones involved. The subjects have studied and became familiarized with the CMJAM and lower extremity symmetry test before the experiment. Before the experiment, we taught the subjects the technical essentials of the jump test, and let them practice repeatedly to get familiar with the movements. They were educated about proper technical movements through videos and live demonstrations and explanations one week before the September 14^th^ in 2022. The subjects were required to complete a 10-min warm-up exercise including jogging, dynamic stretching of the lower limbs, and vertical jumping exercises before the experiment. The electronic scale with an accuracy of 0.1 kg that came with the force platform table was used to measure weight, and the height meter with an accuracy of 1 cm was used to measure height. Each subject performed three CMJAM jumps (with a 2 min passive rest period between each jump) and a lower extremity symmetry test on a force platform table, all of whom were asked to complete as hard as possible.

In the CMJAM test, the athletes stood with a straight torso and the knees fully extended with the feet shoulder-width apart. Athletes kept their hands on their hips throughout the whole jump. They were instructed to perform a quick downward movement and afterward a fast-upward movement to jump as high as possible.

In the lower limb symmetry test, athletes stood on a 30 cm-height jumping platform that induces better jumping motor performance (Prieske et al., 2019). At the command of the evaluator, athletes stepped forward and naturally descended on the force platform. When the foot touched the force platform (moment of contact), athletes immediately performed a single-leg upward jump with the knee slightly turned out, and the body took off vertically with maximum speed and force to reach the maximum height (moment of flight). The same process was carried out by the other side of the lower limb according to the first left and then right jumping order, alternating left and right to complete three times.

The test was performed by the same experimenter with the iPhone 13 ProMax facing the subject (the front), 1.5 m far from the force platform (where the subject landed after each jump), and zooming in on the subject’s feet. Videos were taken indoors under normal incandescent lighting, the iPhone 13 ProMax was fixed on a tripod 30 cm above the ground, as shown in Supplemental Materials. Both the CMJAM and lower limb symmetry tests were completed in the same day. The fencers completed three times CMJAM tests followed by three lower limb symmetry tests, and the swimmers and divers participated in only three times CMJAM tests. The standard of jumping and landing was full extension of the knee and ankle in the same position, and subjects were asked to maximize the height of the jump (and minimize contact time with the ground during the lower extremity symmetry test) while keeping safe. All the test ended at the September 22^ed^ at 2022, and the data was analyzed after a week of the test (Sep. 29^th^, 2022).

### Statistical analysis

#### Statistical analysis of CMJAM test-related data

Descriptive statistics were expressed as $\overline X $ ± sd, and the Shapiro-wilk test was used for normality of the data. The Intraclass correlation coefficient (ICC) was applied to 2-way randomized single measurements (consistency/absolute consistency) (1, 2) to analyze and compare the reliability of the Jump height and Jump flight time measured by the “My Jump 2” application and the force measuring platform. The stability of each subject at the three jumps performed in the test was analyzed using the Cronbach’s alpha test and the coefficient of variation (CV%). The CV% is the typical error of the measurement that expressed as a percentage of the mean. To improve simultaneous validity, the analysis used the highest one jump (29 th jumps), the average of three jumps (29 th jumps) and all jumps (87 th jumps) to obtain better statistical efficacy. Meanwhile, the correlation between the two measures was verified using the Person correlation coefficient (r). Subsequently, the analysis of the ICC was supplemented with Bland-Altman plots to represent the agreement between the two measures (Bland & Altman, 1986), and t-test was used to verify whether there were significant differences between the methods. The statistical significance level was taken as α = 0.05, and the data were processed by SPSS version 26.0 software.

#### Statistical analysis of data related to lower limb symmetry test

The normality of the data was tested by the Shapiro-Wilk test. The t-test was used to compare the mean differences between the two measurements for asymmetry, flight time, and contact time of measurement. As the intra-group correlation coefficient (ICC) was commonly used to assess the consistency or reproducibility of quantitative measurements of the same quantity measured by different systems (Indrayan & Chawla, 1994), we chose this method when analyzing the data related to lower limb symmetry. Meanwhile, the Pearson correlation coefficient was used to analyze the data correlation. Supplying the analysis of intra-group correlation coefficient, BlamD-Altman plot was used to show the consistency of the two methods. The statistical significance level was taken as α = 0.05, and the data were processed by SPSS version 26.0 software.

## Results

### CMJAM test-related data results

**Figure 1:**
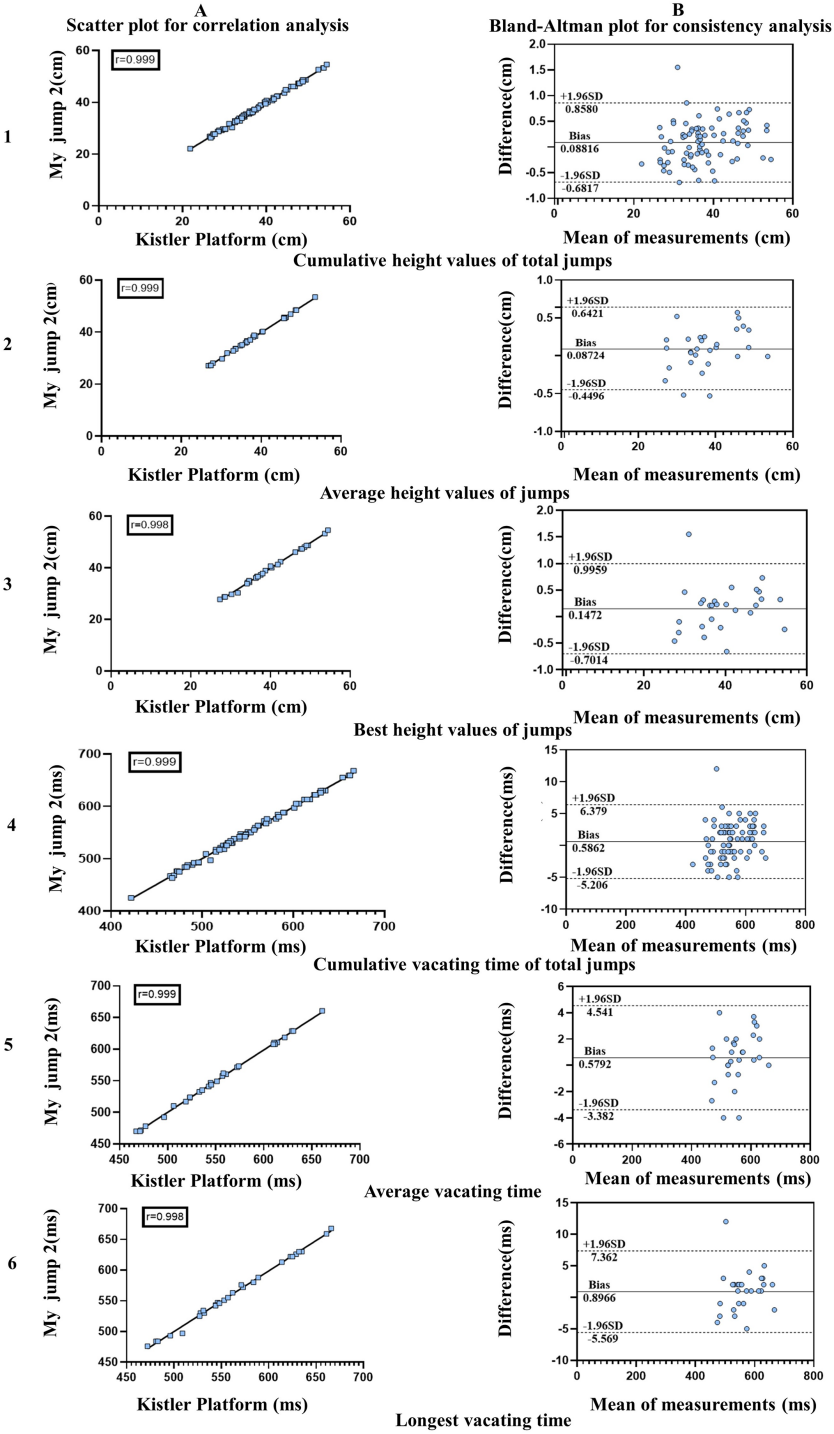
Consistency and correlation between the “My Jump 2” application and the Kistler force gauge in the CMJ longitudinal jump test.

A high correlation was found between the best jump height value (r = 0.999; *P* = 0.001) (Fig. 1A3), the average of the three jump heights (r = 0.999; *P* = 0.001) (Fig. 1A2) and the cumulative height value of the total number of jumps (r = 0.999; *P* = 0.001) (Fig. 1A1) obtained from the “My Jump 2” application and the force platform test, respectively. Those parameters (*P* = 0.001) (Fig. 1A1; Table 2) were highly correlated. The difference in the cumulative height value of the total number of jumps between the two testing methods was 0.088 cm, with a range of 0.858 to −0.682 cm (Fig. 1B1). The optimal jump height value varied between the two testing methods by 0.147 cm, with a range of 0.996 to −0.701 cm (Fig. 1B3). The difference in the mean of the jump height between the two testing modalities was 0.087 cm, with a range of 0.624 cm to −0.450 cm (Fig. 1B2) and a high degree of consistency observed for all three of these metrics obtained by both measurement modalities. Measurements of the obtained vacating time metrics, the agreement between the two measurement methods was the same as above, with a high correlation between the longest time (r = 0.999; *P* = 0.001) (Fig. 1A6), the average time of three jumps (r = 0.999; *P* = 0.001) (Fig. 1A5) and the cumulative time of total jumps (r = 0.999; *P* = 0.001) (Fig. 1A4). There was also a highly correlated relationship between the total number of jumps and the cumulative time to air (r = 0.999; *P* = 0.001) (Fig. 1A4), with a difference of 0.0005862 s between the two testing methods, ranging from 0.006379 s to −0.005206 cm (Fig. 1B4). The difference in the longest time to vacate between the two test methods was 0.0008966 s, with a range of 0.007362 to −0.005569 s (Fig. 1B6). The average time to vacate varied between the two test methods by 0.0005793 s, with a range of 0.004541 to −0.003382 s (Fig. 1B5), all of which showed a high degree of consistency. On the other hand, the t-test showed a significant difference between the two methods when measuring all heights and the first jump height between the different testing methods (*P* < 0.05), while the rest of the methods showed no significant difference. In addition, the second jump had the highest average jump height and the longest jump flight time among the three jumps.

**Table 2:** Jump height values and vacation time recorded by the two measurement methods.

Movement parameters	Kistler platform	“My Jump 2” app	Student-t (*P*)	Person’s r (*P*)
Total number of jumps cumulative height value (cm)	37.88 ± 7.39	37.79 ± 7.29	0.039*	0.999 (0.001)
Jump height average (cm)	37.88 ± 7.28	37.79 ± 7.19	0.097	0.999 (0.001)
Best jump height value (cm)	39.30 ± 7.54	39.16 ± 7.47	0.080	0.998 (0.001)
First jump height value (cm)	37.28 ± 7.53	37.13 ± 7.35	0.043*	0.999 (0.001)
Second jump height value (cm)	38.37 ± 7.74	38.24 ± 7.64	0.160	0.998 (0.001)
Third jump height value (cm)	37.99 ± 7.10	38.00 ± 7.01	0.983	0.999 (0.001)
Total number of jumps cumulative time to vacate (ms)	553.2 ± 54.0	552.6 ± 53.3	0.075	0.999 (0.001)
Average time to vacate (ms)	553.2 ± 53.2	552.6 ± 52.5	0.134	0.999 (0.001)
Longest vacating time (ms)	563.7 ± 54.0	562.8 ± 53.5	0.177	0.998 (0.001)
First jump vacating time (ms)	548.7 ± 56.0	547.6 ± 54.6	0.058	0.999 (0.001)
Second jump vacating time (ms)	556.7 ± 56.1	555.8 ± 55.9	0.152	0.998 (0.001)
Third jump vacating time (ms)	554.3 ± 51.6	554.5 ± 51.0	0.736	0.999 (0.001)

**Note:**

P.s: * *P* less than 0.05, my jump 2 compared with Kistler platform.

The “My Jump 2” application and the force platform were almost identical between the jump height values (ICC = 0.998–0.999, *P* < 0.001) and the time of flight in the air (ICC = 0.998–0.999, *P* < 0.001) obtained from CMJAM measurements in all subjects with high reliability (Table 3). In addition, the consistent results were also obtained when analyzing the intra-group correlation coefficients of people with different sexes and different sports.

**Table 3:** Results of the intra-group correlation coefficient analysis for the CMJAM test of flight time to lift-off and jump height by gender and item.

Movement parameters	Group	“My Jump 2”	Kistler platform	ICC (95%CI)	*P*
Jump height	All people	37.79 ± 7.29	37.88 ± 7.39	0.999	0.001
	Male	41.17 ± 7.33	41.37 ± 7.38	0.999	0.001
	Female	33.63 ± 4.64	33.58 ± 4.67	0.998	0.001
	Fencing	40.24 ± 7.18	40.29 ± 7.45	0.999	0.001
	Swimming team/diving team	36.06 ± 6.92	36.17 ± 6.92	0.999	0.001
Flight time to lift-off	All people	552.6 ± 53.3	553.2 ± 54.0	0.999	0.001
	Male	577.1 ± 52.4	578.5 ± 52.6	0.999	0.001
	Female	522.5 ± 36.8	522.0 ± 37.1	0.998	0.001
	Fencing	570.5 ± 52.4	570.8 ± 54.4	0.999	0.001
	Swimming team/diving team	540.0 ± 50.8	540.8 ± 50.8	0.999	0.001

The “My Jump 2” application showed high stability in the three jump heights (α = 0.999) and flight times (α = 0.999) obtained in the CMJAM test (Table 4), and the Cronbach’s alpha coefficients were greater than 0.8 for all populations, for different genders, and for different items. The CV% of the CMJAM test was less than 10%, but the CV% of the jump height in the CMJAM test was relatively high, which was considered to be due to the different arm swinging patterns of the subjects when they jumped.

**Table 4:** Jump height and time of flight reliability results of the CMJAM test for the “My Jump 2” application and force table.

Movement parameters	Group	My Jump 2			Kistler platform		
		Intrasession		Intersession	Intrasession		Intersession
		α	CV%	r	α	CV%	r
Jump height	All people	0.999	19.3	0.999	0.999	19.5	0.999
	Male	1.000	17.8	0.999	0.999	17.8	0.999
	Female	0.998	13.8	0.994	0.999	13.9	0.994
	Fencing	0.999	17.8	0.999	0.999	18.5	0.999
	Swimming team/diving team	0.999	19.2	0.999	0.999	19.1	0.999
Flight time to lift-off	All people	0.999	9.7	0.999	0.999	9.8	0.999
	Male	1.000	9.1	0.999	1.000	9.1	0.999
	Female	0.998	7.1	0.998	0.998	7.1	0.998
	Fencing team	0.999	9.2	0.999	0.999	9.5	0.999
	Swimming team/diving team	0.999	5.5	0.998	0.999	5.4	0.998

**Note:**

P.S.: α, Cronbach’s alpha; CV%, Coefficient of variation; r, Pearson correlation coefficient.

### Results of data related to lower limb symmetry test

**Figure 2:**
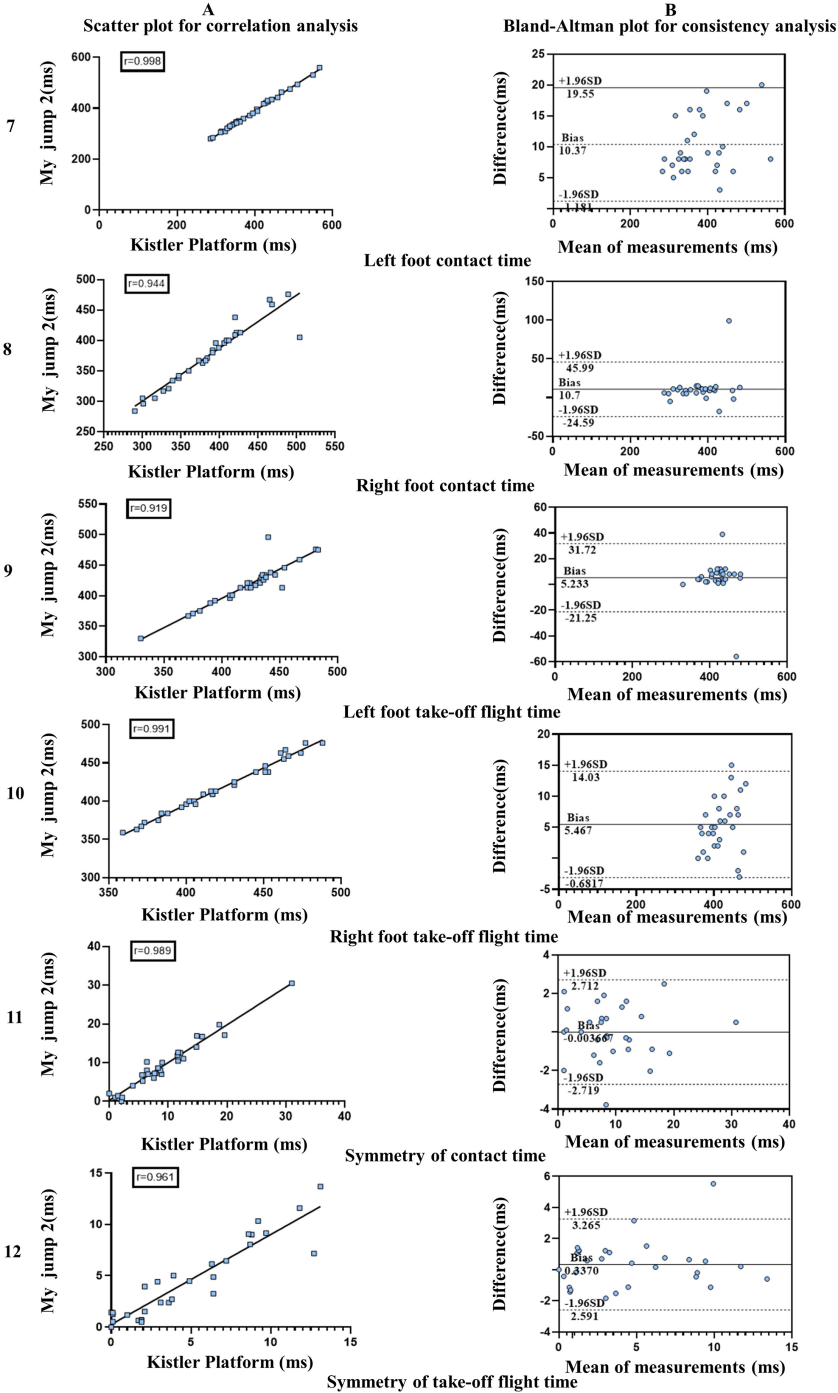
Consistency, correlation between the My Jump app and the force table in lower extremity symmetry testing.

The “My Jump 2” application and the force platform test obtained in the symmetry test for the left foot contact time (ICC = 0.998–1, *P* = 0.001), right foot contact time (ICC = 0.937–0.985, *P* = 0.000), left foot take-off flight time (ICC = 0.912–0.980, *P* = 0.001), right foot take-off flight time (ICC = 0.992–0.998, *P* = 0.001), symmetry of contact time (ICC = 0.976–0.994, *P* = 0.001), and symmetry of take-off flight time (ICC = 0.921–0.982, *P* = 0.001) were highly consistent with each other (Table 5). In the lower limb symmetry test, the r values of Pearson correlation index for both measurement methods were greater than 0.9, but significant differences (*P* < 0.05) could be observed in the contact time and flight time indexes after t-test, while the t-test indexes of contact time symmetry and flight time symmetry were diametrically opposed to the former and differed less (*P* > 0.05), so it was concluded that in the symmetry indexes, the two methods were not significantly different, and the Bland-Altman plot results also showed a high degree of agreement (Fig. 2).

**Table 5:** Results of lower limb symmetry data measured by the “My Jump 2” application and the Kistler platform.

	My Jump 2	Kistler platform	ICC (95%CI)	Correlation analysis r (*P*)	Student t		My Jump 2	Kistler platform	ICC (95%CI)	Correlation analysis r (*P*)	Student t
	Left foot contact time (ms)						Right foot contact time (ms)				
All people	383.4 ± 72.0	393.8 ± 73.7	0.994	0.998 (0.001)	0.001		376.1 ± 50.3	386.8 ± 54.6	0.96	0.944 (0.001)	0.001
	Left foot flight time (ms)						Right foot flight time (ms)				
All people	418.5 ± 34.2	423.8 ± 32.7	0.953	0.919 (0.001)	0.001		416.9 ± 35.3	422.3 ± 36.5	0.991	0.993 (0.001)	0.001
	Contact time symmetry (%)						Time-of-flight symmetry (%)				
All people	9.41 ± 6.46	9.41 ± 6.47	0.989	0.941 (0.000)	0.989		4.80 ± 4.02	4.46 ± 3.80	0.961	0.923 (0.001)	0.381

## Discussion

The purpose of this study is to analyze the validity and reliability of the “My Jump 2” application in measuring the CMJAM test (jump height, flight time) and lower limb symmetry test parameters (left and right foot contact time, left and right foot flight time, contact symmetry, flight time symmetry) in athletes of different sports. The main results showed that the relevant motion parameters of CMJAM and lower limb symmetry tests measured by using the “My Jump 2” application were in good agreement with the results obtained from the force bench and had a high degree of correlation. The “My Jump2” application was found to be a highly valid and reliable tool for measuring jumping performance such as the CMJAM and lower limb symmetry tests, with high daytime test reliability for both male and female athletes in many different sports such as fencing, swimming and diving.

According to the literature, the “My Jump 2” application has not been used to evaluate jump performance metrics on the CMJ test and lower limb symmetry test among the fencers and water-project sports (swimmers or divers) in previous studies. The average difference in CMJ vertical jump height between the “My Jump 2” application and the ergometer in this study was only 0.1 cm, which was slightly lower than the average difference between devices obtained by Gallardo-Fuentes et al. (2016) when evaluating the CMJ of male and female athletes (0.20 cm) and the average difference obtained in the population of elementary school students studied (0.20–0.30 cm) (Bogataj et al., 2020). In addition, the mean differences in CMJ results between the two measurement devices obtained in another study on a population of adolescent athletes were 0.59 cm (Rogers et al., 2019), which was slightly higher than the mean difference result obtained in our study (0.1 cm) again. Balsalobre-Fernández, Glaister & Lockey (2015) found a higher average difference result (1.1–1.3 cm). However, this may be related to the lower sampling frequency of the iPhone camera applied (iPhone 5 s; 120 Hz), as the lower video recording/capture frequency may increase the probability of not correctly identifying the “take-off moment”. Instead, the iPhone 13 ProMax applied in this study used a 240 Hz sampling frequency camera, which would greatly reduce the disadvantage of insufficient sampling frequency and increase the accuracy of motion classification. A study that comparing portable measurement devices and force benches showed the average difference in CMJ jump height was between −1.06 and 11.7 cm (Buckthorpe, Morris & Folland, 2012; Choukou, Laffaye & Taiar, 2014; Glatthorn et al., 2011). The average difference between the two tools from different literature may be the difference in sampling rate and performance level. Our research supports the above results that “My Jump 2” could capture jump heights with an average difference of 1 cm or less on a device that captures video at 240 Hz. However, the coefficients of variation for jump height (13.8–19.5%) and for time to vacate in this study ranged from 5.4–9.8%, both of which were much higher than those in the CMJ test-related studies mentioned above, for example, the coefficients of variation ranged from 4.63–7.6% in a study on CMJ vertical jump height in young athletes(Bishop et al., 2022). And in the elementary school, the coefficient of variation associated with CMJAM in the population was even lower, with a range of 3.9–4.9%(Bogataj et al., 2020). Although the coefficient of variation in studies related to the older population (10.096–10.111%) was higher (Cruvinel-Cabral et al., 2018), it was still lower than the results obtained in the present study (19.3–19.5%). Furthermore, CMJAM can be a confounding variable because the shoulder, elbow, hip and ankle muscles work together and the body coordination during jumping may have an impact on jumping performance (8–11%) (Hara et al., 2008; Lees, Vanrenterghem & De Clercq, 2004). It is hypothesized that this result may be the difference in proficiency in CMJAM jumping techniques in different sports, and the relatively large difference in the age and training years of the athletes can also be a potential factor in this result.

The average differences in the flight time of CMJ was 3 ms, which was slightly lower than the average differences between the left and right foot vacations in the lower limb symmetry test (5 ms). This may be due to the ability of the athletes to master the technical movements of the CMJ longitudinal jump more skillfully leading to this differential result. And we found that most of the athletes were more proficient in the CMJ vertical jump technique during the test as the athletes took explosive training of the lower limb vertical jump in the daily training process frequently compared to the lower limb symmetry test. When Barbalho et al. (2021) assessed the asymmetry of interlimb jumping in young soccer players, the mean difference in contact time was 9.9 ms for the left foot and 8.9 ms for the right foot, both of which were slightly smaller than the mean differences in contact time between the left and right foot in the present experiment (left: 10 ms, right: 11 ms). In terms of flight time, the mean difference in the left foot take-off time in this study was 7.1 ms, the right foot take-off time was 4.5 ms, and the average difference between left and right foot take-off was 5 ms. The average difference in symmetry of contact time for the lower extremity symmetry test in this study was only 0.004%, much lower than the 0.7% in previous studies. However, the average difference in symmetry of take-off flight time was 0.337%, slightly higher than the average difference in previous studies (0.1%) (Barbalho et al., 2021). The above data revealed that our study was in general agreement with the results of Matheus-Barbalho’s study, but the average difference in the above data is slightly lower overall than previous studies, which maybe the different technical characteristics of sports, such as the different dominant foot and kicking postures of soccer players and the unilateral limb dominance of fencing and other asymmetrical sports. Besides, the different sampling frequency of different types of force measuring stations will also have an influence. The previous studies have demonstrated a difference of approximately 1.0 cm in the measurement of vertical jumping movements on infrared platforms compared to 1,000 Hz force platforms (Choukou, Laffaye & Taiar, 2014). Moreover, it showed that the average difference between the results of the acceleration measurement system (Mootest SA, Rolle, Switzerland) at 1,000 Hz and the results of the force platform was 3.6 cm (Buckthorpe, Morris & Folland, 2012), which is higher than the “My Jump 2” application in this study. The high-speed camera is the most accurate device for measuring longitudinal jump height besides the force table (Balsalobre-Fernández et al., 2014; García-López et al., 2005). The professional high-speed camera records at a sampling rate of 1,000 Hz, and compared with a 1,000 Hz force measurement table, the difference in the take-off flight time is only 1.3 ms (Requena et al., 2012). Therefore, compared with the expensive devices mentioned above, the CMJ measured by “My Jump 2” is with essentially the same accuracy (about 3 ms of flight time or 0.1 cm of jump height). The CV% of the MARS system employing the Kistler force table in this study (5.9 ± 4.1%) (Hébert-Losier & Beaven, 2014) was higher than the Optojump system (2.7%) used in other studies (Choukou, Laffaye & Taiar, 2014), and the different reference device may be another important factor that can influence the difference in the mean differences between the results of this study and those of previous studies.

It has been reported that well-developed muscle strength and power levels play an important role in achieving a high level of swimming performance. The time of swimming jumping period accounts for 30% of the total race time (Cossor, Blanksby & Elliott, 1999), and the shorter the distance, the more important an explosive start into the water becomes. West et al. (2011) showed that a successful swimming jump into the water depended on many factors, including reaction time, vertical and horizontal forces generated by the lower limb muscles during the push-off phase of the obstacle, and low resistance during the underwater glide phase. Therefore, the increase in the maximum vertical jump height in swimming athletes is usually accompanied by a better competitive performance. It has been suggested that the “My Jump 2” application may be well suited to evaluate athletes who participate in sports with explosive power.

The characteristic of fencing is the repeated attack with the “fencing lunge”. In the lunge “down-thrust” movement, the athlete is often burdened with large centrifugal forces from the front lunge support leg (as it absorbs the force of the sprinting movement) and higher propulsive forces from the backstroke extension leg (Turner et al., 2014). The nature of the sport dictates that fencers always use the dominant leg in conjunction with the dominant hand. Therefore, there could be interlimb asymmetry and the fencer’s dominant leg may affect their lower limb symmetry. The literature showed an increased incidence of injuries in non-athlete and athlete groups with interlimb asymmetry was more than 15% (Barber et al., 1990; Grindem et al., 2011; Impellizzeri et al., 2007), and the interlimb asymmetry less than 10% has been proposed as a goal for athletes returning to sport (Kyritsis et al., 2016). In this study, by analyzing the time of flight symmetry and time of contact symmetry of the lower extremity limb asymmetry test in fencers, the average value of contact time symmetry was 10.11% in male fencers, while 8.73% in females, which implied that lower extremity symmetry of most subjects was close to or even above the threshold for a negative impact on jumping performance but below the threshold for an increased risk of injury. It was similar to the conclusions reached in the previous article.

Therefore, the specially developed iPhone application “My Jump 2” is valid and reliable and can be used to measure different metrics such as jump height, flight time, and contact time during rapid stretch-shortening cycle muscle movements (*i.e*., CMJ vertical jump) and symmetrical lower limb jump tests for athletes of different sports and genders, including fencing, swimming, diving, and track and field soccer. The coaches can easily and quickly monitor an athlete’s vertical jumping ability and assess the athlete’s explosive lower body strength level by this software.

## Conclusion and suggestion

The “My Jump 2” application is a highly valid and reliable tool for measuring jumping performance such as CMJAM and lower limb symmetry tests, with high daytime test reliability for both male and female athletes in many different sports such as fencing, swimming and diving. The camera sampling frequency is related to the jump height error, and the 240 Hz device is beneficial to reduce the error.

Beginner users who lack a large number of experience analysis in motion video analysis can also use “My Jump 2” App to analyze jumping movements and get stable measurements. The coaches, trainers and athletes are encouraged to incorporate the app and testing methods into their daily fitness test evaluations to prevent sports injuries and improve the rehabilitation process or athletic performance. In addition, due to the portability and utility of smartphone, it will soon become a common tool for measuring physical properties with a high degree of accuracy.

## Supplemental Information

10.7717/peerj.17658/supp-1 Supplemental Information 1 Take-off and landing phase frames on the My Jump 2 app.

10.7717/peerj.17658/supp-2 Supplemental Information 2 Original data.

## Additional Information and Declarations

### Data Availability

The following information was supplied regarding data availability: The raw data is available in the Supplemental File.
