# Supplementary figures and images for: Reliability and validity of “My Jump 2” application for countermovement jump free arm and interlimb jump symmetry in different sports of professional athletes
Source: PeerJ. 2024 Jul 9;12:e17658. doi: 10.7717/peerj.17658 (PMC11244033; doi:10.7717/peerj.17658)

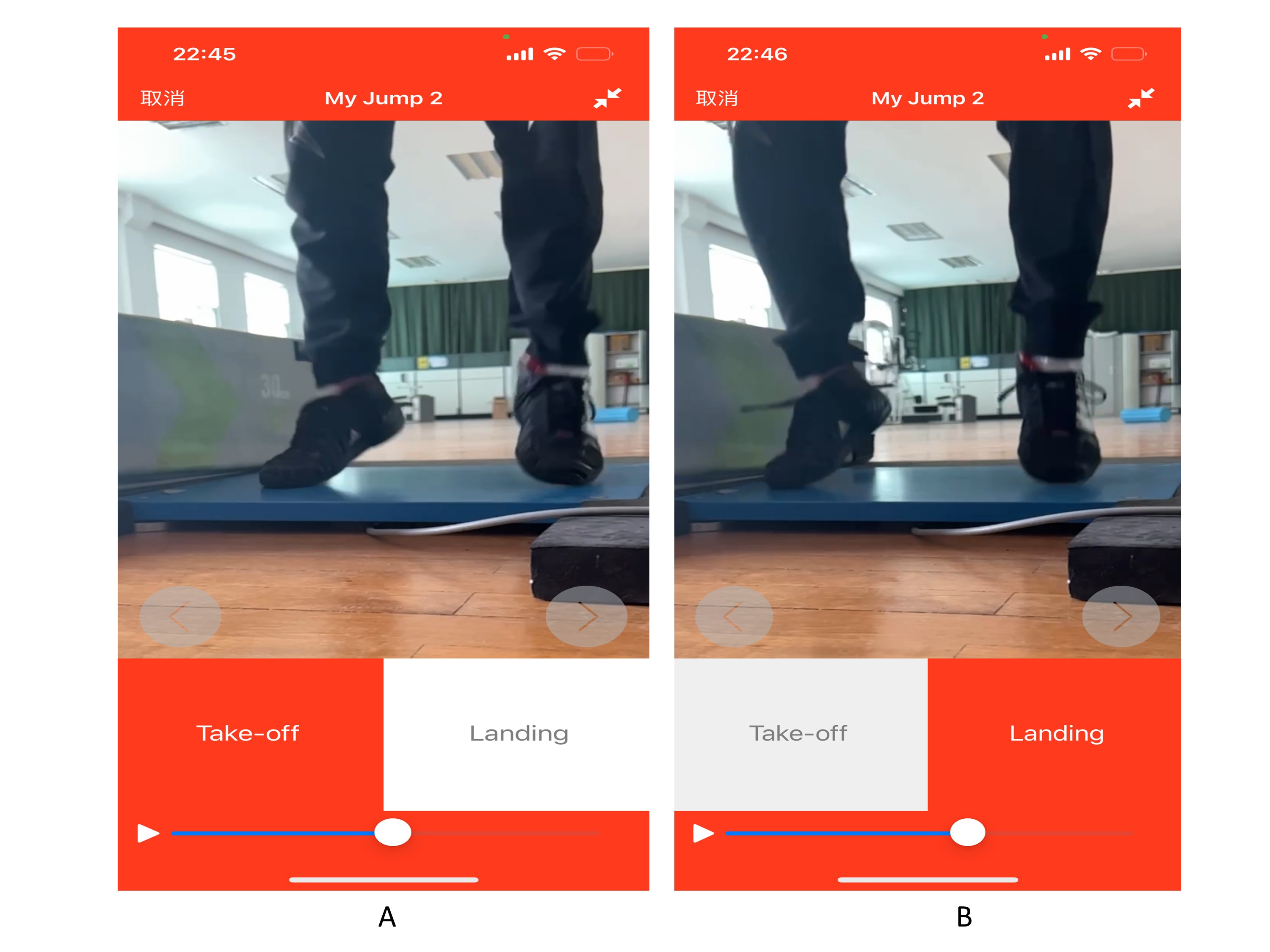

Supplement: Supplemental Information 1 [file peerj-12-17658-s001.jpg]
